# Experimental and simulation-based investigation of He, Ne and Ar irradiation of polymers for ion microscopy

**DOI:** 10.3762/bjnano.7.104

**Published:** 2016-08-02

**Authors:** Lukasz Rzeznik, Yves Fleming, Tom Wirtz, Patrick Philipp

**Affiliations:** 1 Advanced Instrumentation for Ion Nano-Analytics (AINA), MRT Department, Luxembourg Institute of Science and Technology (LIST), 41 rue du Brill, L-4422 Belvaux, Luxembourg

**Keywords:** Helium ion microscopy, irradiation, polymers, preferential sputtering, secondary ion mass spectrometry, simulations

## Abstract

Secondary ion mass spectrometry (SIMS) on the helium ion microscope (HIM) promises higher lateral resolution than on classical SIMS instruments. However, full advantage of this new technique can only be obtained when the interaction of He^+^ or Ne^+^ primary ions with the sample is fully controlled. In this work we investigate how He^+^ and Ne^+^ bombardment influences roughness formation and preferential sputtering for polymer samples and how they compare to Ar^+^ primary ions used in classical SIMS by combining experimental techniques with Molecular Dynamics (MD) simulations and SD_TRIM_SP modelling. The results show that diffusion coefficients for He, Ne and Ar in polymers are sufficiently high to prevent any accumulation of rare gas atoms in the polymers which could lead to some swelling and bubble formation. Roughness formation was also not observed. Preferential sputtering is more of a problem, with enrichment of carbon up to surface concentrations above 80%. In general, the preferential sputtering is largely depending on the primary ion species and the impact energies. For He^+^ bombardment, it is more of an issue for low keV impact energies and for the heavier primary ion species the preferential sputtering is sample dependent. For He^+^ steady state conditions are reached for fluences much higher than 10^18^ ions/cm^2^. For Ne^+^ and Ar^+^, the transient regime extends up to fluences of 10^17^–10^18^ ions/cm^2^. Hence, preferential sputtering needs to be taken into account when interpreting images recorded under He^+^ or Ne^+^ bombardment on the HIM.

## Introduction

Progress in materials and life sciences requires sample characterisation with high lateral resolution and high sensitivity. A technique which allows for both is secondary ion mass spectrometry (SIMS). Combined with a high-resolution mass spectrometer, mass interferences can be avoided and isotopes and small cluster ions identified unambiguously. These properties have been used since the early days of SIMS for imaging applications [1]. On the Cameca NanoSIMS, which is a SIMS instrument dedicated to high-resolution imaging, a lateral resolution of around 50 nm can be reached. Recently, the development of a SIMS add-on system for the helium ion microscope (HIM) [2] demonstrated SIMS imaging with even higher lateral resolution in the 10 nm range [3]. Initially the HIM has been developed for high resolution electron microscopy and nanofabrication using the He^+^ or Ne^+^-emitting atomic level ion source (ALIS) [4]. Compared to cluster ion bombardment, the use of monoatomic primary ion species (such as Cs^+^, O^−^, Ga^+^) for imaging in SIMS allows significantly higher lateral resolutions to be achieved. However, fragmentation occurring under such monoatomic primary ion irradiation implies a loss of molecular information for organic and biological samples. This can be partially compensated by the use of isotopically labelled molecules.

In life sciences, imaging with monatomic primary ion beams has already been applied to many problems. Typical examples include the investigation of chromosomes [5], the localization of arsenic [6] and iodine [7] in human hair, precise localisation of elements at sub-cellular level in cell biology [8], study of uptake processes in microbiology [9] and plant biology [10–11]. In studies of this kind, elemental mapping by dynamic SIMS has the advantage of high lateral resolution and better chemical sensitivity than many other techniques.

To get valuable information from samples, it is important to control the interactions between primary ions and the samples. Numerical simulations are one tool which can provide important information about particle–surface interactions, both for inorganic [12–15] and organic samples [16–19]. For the analysis of organic matter, information on preferential sputtering and the modification of the sample under ion bombardment is obtained. As an example, one may cite molecular dynamics (MD) simulations of Ar^+^ irradiation on a benzene overlayer on a Ni(001) surface [17] and on an ethylidyne (C_2_H_3_) overlayer on a Pt(111) substrate [18].

Ion bombardment on polymers was reported first for 1 keV Ar bombardment on polyethylene [19]. Both single impacts and 5 consecutive impacts were carried out with an incidence angle of 60° on systems with 52 Å × 49 Å × 42 Å and 37 Å × 40 Å × 42 Å, respectively. This corresponds approximately to a fluence of 3 × 10^13^ atoms/cm^2^. The ion bombardment causes chain scission, cross-linking as well as carbonization of the target. For the multiple impacts, larger molecular fragments (up to C_36_H_69_) and a smaller H to C ratio was observed. These results show that different species are not necessarily sputtered in the ratio as present in the sample, which may lead to the enrichment of one of the elements in the near-surface region. This is known as preferential sputtering. This issue was also studied by Hnatowicz et al. for 100 keV He^+^, Ne^+^, Ar^+^ and Kr^+^ bombardment of polyimide and the depletion of H and O for fluences above 10^14^ ions/cm^2^ [20]. More recent studies on monatomic bombardment on polymers report on 5 keV and 500 eV Ar atom bombardment of polystyrene oligomers [21] and polystyrene molecules containing 61 repeat units [22] adsorbed on Ag(111), respectively. In the first study, the excited volume in the substrate induces collective motion which allows molecular fragments to desorb.

The degradation of organic molecules and their fragmentation are not the only important parameters for imaging in SIMS using light primary ions. For inorganic matter, the swelling of samples under ion bombardment has been observed for He^+^ and Ne^+^ bombardment of Si for fluences exceeding 10^17^ ions/cm^2^ [23]. Usually, analysis conditions in HIM are optimized to avoid such problems [24]. Sample damaging is also an issue in TEM sample preparation, which HIM might be used for.

In this work, we are going to invest the degradation of polymer samples and the formation of secondary ions under helium and neon bombardment for conditions used for SIMS imaging on the helium ion microscope (HIM). Of particular interest is how the sputtering processes differ from irradiation with heavier and chemically inert ions used in SIMS (e.g., gallium and argon). Therefore He^+^ and Ne^+^ bombardment will be compared to Ar^+^ bombardment for several polymers and several impact energies. The objective is to identify possible artefacts related to the sputter process and to control them. The study relies on experimental work and numerical simulations making use of the SD_TRIM_SP [25], which allows for higher fluences than MD simulations.

## Results and Discussions

### AFM measurements of irradiated areas

**Figure 1:**
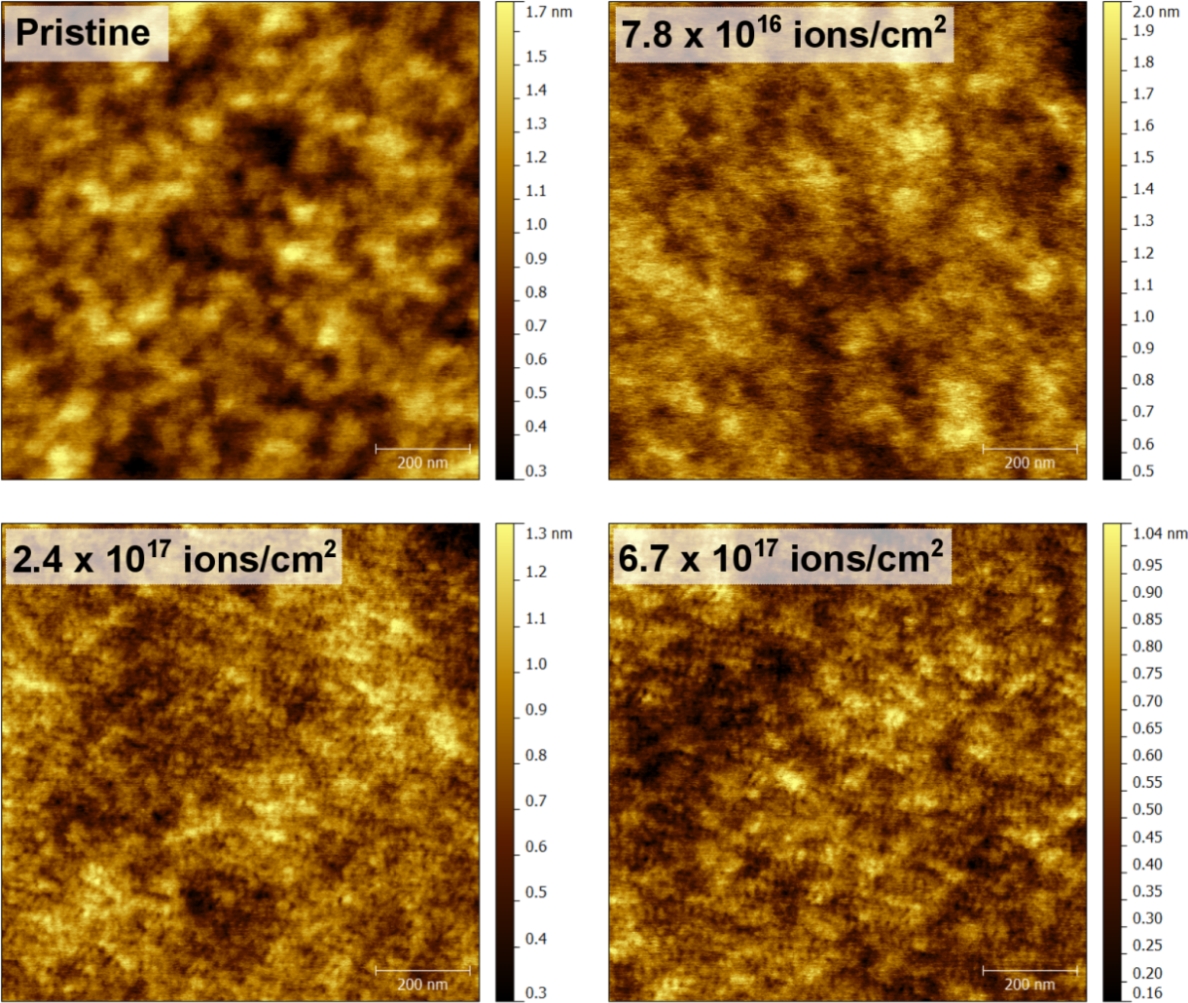
Changing topography of PMMA bombarded by 5.5 keV Ne^+^ as a function of fluence (FOV: 1 × 1 µm^2^).

An example of the changing topography with respect to primary Ne^+^ fluence is shown in Figure 1 for PMMA. No significant roughness with amplitudes higher than 1 nm is developing, in contrast to what is observed for inorganic samples [26–27]. Only a change in the surface structure from a larger to a finer grain structure is observed. At this point it is not clear whether these structures are related to some degradation of the polymer materials or whether they are due to bubble formation induced by the implantation of rare gas atoms. The latter has been observed among other for gallium nitride [28], silicon [29], iron [30] or steel [31], and tungsten [32] under helium irradiation.

**Figure 2:**
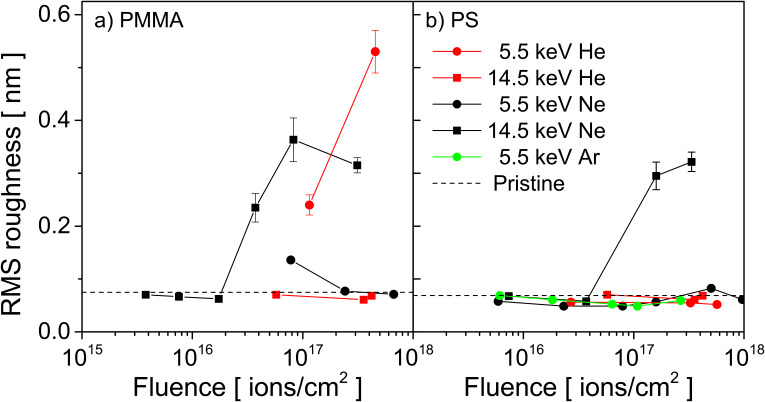
RMS roughness changing with primary He^+^ and Ne^+^ fluence when bombarding (a) PMMA and (b) PS.

The evolution of roughness for Ne^+^ irradiation of PMMA can be better seen in Figure 2a where the value of RMS roughness does not exceed 0.4 nm. The same is true for He^+^ irradiation where the RMS roughness stays below 0.55 nm (Figure 2a). The same experiments were carried out for PS. For He^+^, Ne^+^ and Ar^+^ bombardment, no major increase of the RMS is observed (Figure 2b), similar to the results obtained on PMMA. This shows that roughness formation does not seem to be a major issue for polymers under rare gas irradiation, opposite to what is observed for inorganic materials where the implantation of the rare gas species leads to irradiation-induced roughness formation, swelling and bubble formation. To understand how and why the behaviour of polymers under rare gas irradiation differs from inorganic materials, numerical simulations have been carried out to study the diffusion behaviour of these species in polymers, the degradation of polymers under rare gas ion bombardment as a function of fluence and the implantation of He^+^, Ne^+^ and Ar^+^ primary ions into the polymer samples. Diffusion coefficients obtained from the first study carried out by MD simulations will be used in work on irradiation damage and implantation.

### Diffusion of rare gases in polymers

**Figure 3:**
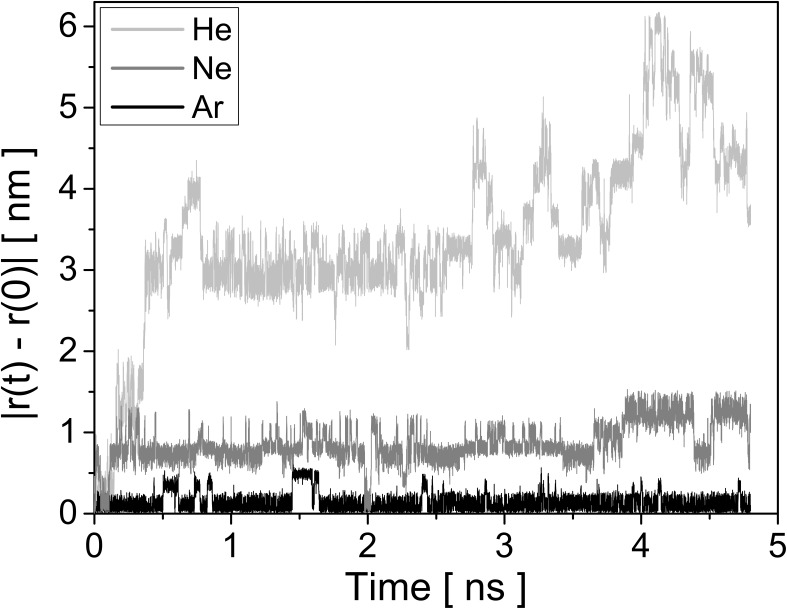
Displacement of He, Ne and Ar from their initial positions in HD-PMMA sample at 300 K. No periodic boundary conditions were used to extract atoms positions from trajectory file, that is why the maximum displacement for He atom extends above the system size. The trajectories have been selected to reflect the average mobility of the rare gas species in HD-PMMA.

The mobility of noble gas atoms in polymer samples has been studied by MD simulations in order to explain previous results and to get some input data for simulations on the irradiation of polymers with rare gas atoms. The motion of the diffusing atoms in the polymer sample is traced by following the displacement from their initial position 
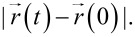
 Figure 3 shows the displacement of He, Ne and Ar in HD-PMMA. Several features can be seen in the plot. Small oscillations can be seen for all curves. They are due to the fact that not the whole volume is accessible for the rare gas atoms and repulsive interactions between the polymer atoms and He, Ne or Ar restrict the volume accessible to the latter to relatively small voids in which they are somehow imprisoned and oscillate around their equilibrium position. The size of the voids depends on the polymer type and density, and the alignment of the molecules. The size of the rare gas atoms also matters. For Ar the amplitude of oscillations is around 0.25 nm, which is significantly smaller than the Ne and He values of 0.5 nm and 1 nm at maximum. Hence, the larger Ar atom is more effectively imprisoned inside the polymer, which is not surprising. The size of the atom seems also to limit the amplitude of oscillations inside one civility. However, from time to time species can jump to another void, changing significantly the distance from their initial position. Once more, the smaller He atoms find more open connections between voids, making longer and more frequent jumps compared to the heavier Ar atoms. The behaviour of Ne is in between.

**Figure 4:**
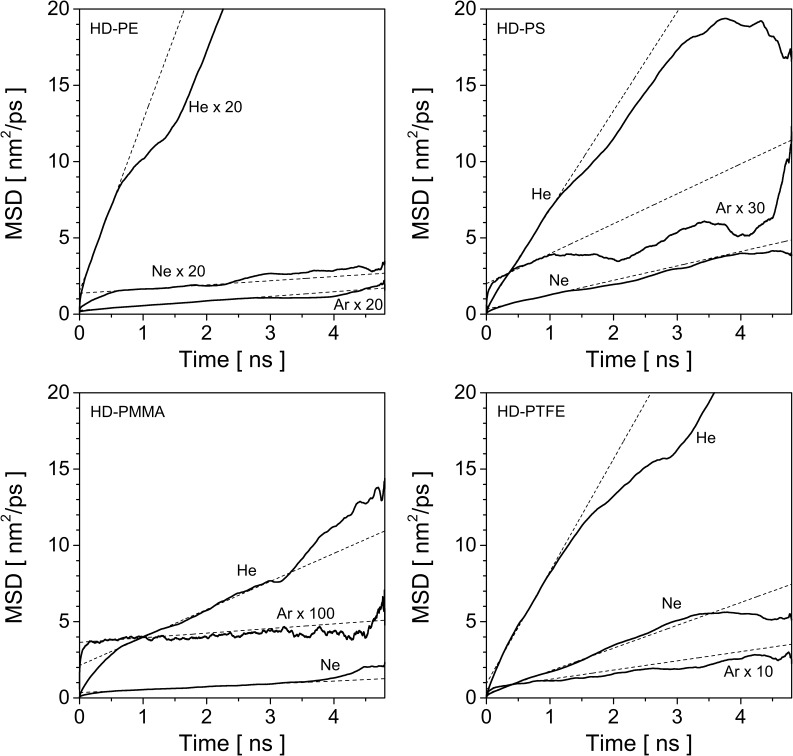
Mean square displacement (MSD) of helium, neon and argon at 300 K in a) HD-PE, b) HD-PS, c) HD-PMMA and d) HD-PTFE. For HD-PE all curves have been scaled up by a factor 20, for other polymers the curves for argon have been scaled up by a factor 10, 30 or 100 as is depicted on the each graph. The dashed lines show the linear fits to the plots.

The displacement of the noble gas atoms inside the polymer samples is described using the mean square displacement (MSD). Their trajectories have been recorded with a time step of *dt* = 0.2 ps, which makes 24000 trajectory points during the last 4.8 ns of the simulation. For each polymer sample, the MSD is then averaged over all diffusing atoms of a given species. The calculated MSD for He, Ne and Ar in HD-PE, HD-PS, HD-PMMA and HD-PFTE are shown in Figure 4. The linear regime in the MSD lasts for 2–3 ns depending on the polymer. For this time range, the diffusion coefficient *D* can be calculated by making a linear fit to the MSD curve, MSD(*t*) = 6*Dt* + *b* [33]. For longer times the MSD starts to behave unpredictably due to reduced sampling leading to statistical fluctuations. The calculated diffusion coefficients are summarized in Table 1 together with the available reference values.

**Table 1:** Summary of the diffusion coefficients (× 10^6^ cm^2^ s^−1^) of He, Ne and Ar in PE, PS, PMMA and PTFE calculated in this work with comparison to experimental and other simulation studies.

Diffusing atom	Polymer	Density [g/cm^3^]	Diffusion coefficient [× 10^6^ cm^2^ s^−1^]	Reference values from simulations [× 10^6^ cm^2^ s^−1^]	Experimental reference values [× 10^6^ cm^2^ s^−1^]

He	LD-PE	0.92	4.27		2.4 [37] 7.7 [38]
Ne			0.9		
Ar			0.11		0.57 [39]

He	HD-PE		0.93		
Ne			0.14		
Ar			0.025		

He	LD-PS	1.01	20.9	14 [34]	10.4 [40]
Ne			2.95	2.1 [34]	2.4 [41]
Ar			0.31	0.02 [35]	0.24 [41]

He	HD-PS	1.04	10.79	15 [34]	
Ne			1.58	1.6 [34]	
Ar			0.11	0.075 [35]	

He	LD-PMMA	1.17	13.49		
Ne			1.35		
Ar			0.143		

He	HD-PMMA	1.20	3.06		
Ne			0.32		
Ar			0.005	0.001 [42]	

He	LD-PTFE	1.55	326.3		
Ne			122.5		
Ar			47.7		

He	HD-PTFE	2.17	12.28		
Ne			2.49		
Ar			0.1		

Comparing the different MSD curves, or the calculated diffusion coefficients, it can be seen that the mobility of the rare gas atoms increases with decreasing atom size (Figure 4). In general, the diffusion coefficients of He and Ar differ by about 1 to 2 orders of magnitude, depending on the polymer. Comparing the polymers presented in Figure 4, the highest diffusion coefficients are observed in HD-PTFE followed by HD-PS. The diffusion coefficients for HD-PE and HD-PMMA are smaller. For the polymers selected for this comparison, the PS has lower density (1.04 g/cm^3^) compared to PMMA (1.20 g/cm^3^) so a slightly larger mobility of He atoms in PS is in line with expectations. However PTFE is the most dense (2.17 g/cm^3^) of all polymers, so the mobility of He is expected to be smallest in that polymer. On the contrary, the opposite effect is anticipated for PE which has the lowest density (0.96 g/cm^3^). MD simulations give however an opposite trend with He moving more freely in PTFE than in PMMA, PS and even PE. This means connections between voids are more likely in PTFE, allowing He to jump more frequently. As the size of the volume accessible for a diffusing atom is related to sample density and size of the polymer atoms, it can be deduced that F and C in PTFE leave more space to diffusion to He than the atoms in PE, PS or PMMA. And indeed, if we look at the atomic density of the selected samples, PFTE has the smallest (78 atom/nm^3^), with PS (97 atom/nm^3^) and PMMA (109 atom/nm^3^) in the middle and PE (124 atom/nm^3^) with the highest. PTFE is composed of F atom, which is heavier than O or H atoms composing PS and PMMA. So, PTFE has at the same time the largest mass density and the lowest atomic density, which enhances the mobility of rare gas atoms inside this polymer compared to the other three polymers. Similar conclusions can be drawn for the LD polymers used in this work. However, even if the results taken separately for LD and HD polymers allow to identify the atomic density as one driving force behind the difference in atom mobility in the selected polymers, taking all the LD and HD polymer data together does not support this observation as an increasing atomic density does not induce a decreasing diffusion coefficients. This means that both mass and atomic densities play an important role in transport of rare gas atoms in a polymer.

In general, there is also a good agreement between the diffusion coefficients calculated in this work and reference values found in literature (Table 1). Experimental diffusion coefficients are only available for two polymers. For LD-PE, there is a good agreement between experimental and simulation values. For Ar, the experimental value is higher, but the order of magnitude is still good. For LD-PS, there is a factor 2 between the experimental and simulation values. For Ne and Ar, there is almost perfect agreement. For LD-PS and HD-PS, the trend between simulated reference values and our values is identical, suggesting that our values for HD-PS are also valid. For both polymers, the agreement is much better for He and Ne than for Ar, showing the influence of the experimental method on the results. Indeed, values for He and Ne are found in one reference [34] and the one for Ar in another [35]. Hence, the diffusion coefficients for which no reference values exist can also be considered to be reliable and will be used in subsequent numerical simulations on the irradiation of polymers with He^+^, Ne^+^ and Ar^+^ ions. Globally the diffusion coefficients vary also in the range of 10^−4^–10^−9^ cm^2^/s, which is much higher than the diffusion coefficients in inorganic materials (e.g., of the order of 10^−16^ cm^2^/s for He diffusion in Si at room temperature [36]).

### Influence of rare gas outgassing on irradiation results

The afore-calculated diffusion coefficients will be used to describe the diffusion of rare gases in polymers during He^+^, Ne^+^ and Ar^+^ bombardment of the different polymers. Before, we will investigate by SD_TRIM_SP how the diffusion coefficient influences the rare gas concentration in the polymers during ion bombardment. Therefore their values have been changed from 1.67 × 10^−17^ to 1.67 × 10^−17^ cm^2^ s^−1^ . It should be noted that the experimental diffusion coefficients range in between 1.67 × 10^−8^ and 1.67 × 10^−5^ cm^2^ s^−1^.

**Figure 5:**
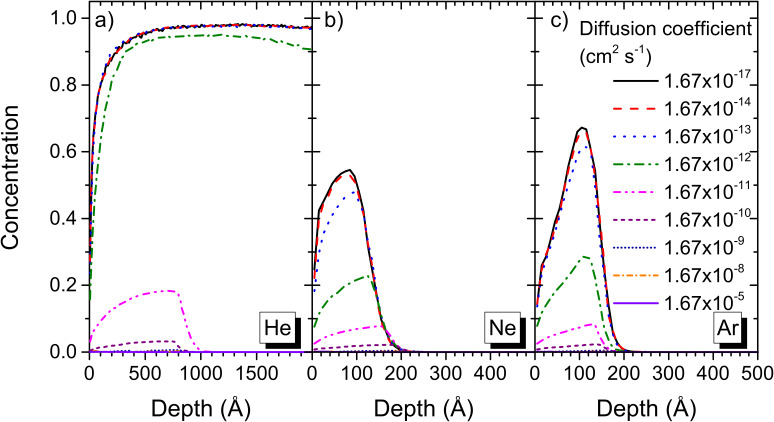
Implantation profiles in polyethylene obtained by SD_TRIM_SP for diffusion coefficient ranging from 1.67 × 10^−17^ to 1.67 × 10^−5^ cm^2^ s^−1^ for a) helium, b) neon and c) argon.

**Figure 6:**
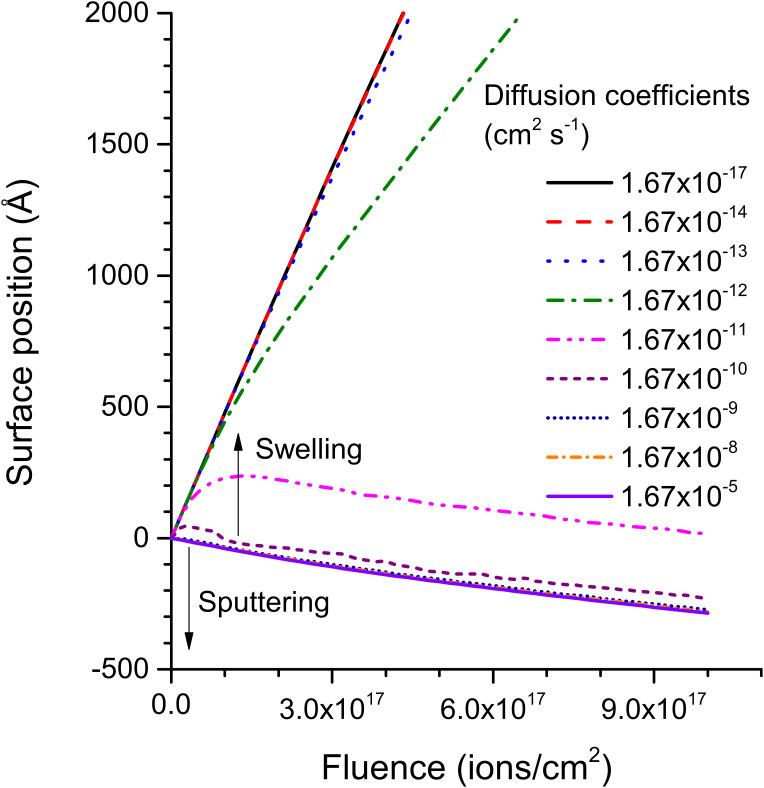
Surface sputtering vs swelling for different diffusion coefficients for helium irradiation of PE.

The simulations show that the implantation profile and concentrations of rare gases inside the sample largely depend on the size of the implanted species (Figure 5). For helium, swelling of the sample is observed for diffusion coefficients up to 1.67 × 10^−12^ cm^2^ s^−1^ (Figure 6). The transition from swelling to sputtering occurs at a diffusion coefficient of 1.67 × 10^−11^ cm^2^ s^−1^. For diffusion coefficients of 1.67 × 10^−11^ cm^2^ s^−1^ and 1.67 × 10^−10^ cm^2^ s^−1^, implantation profiles with maximum concentrations which do not exceed 20% and 3% are observed. For larger diffusion coefficients, the helium concentration drops almost to 0%, i.e., it diffuses almost completely out of the polymer sample. For Ne^+^ and Ar^+^ bombardment, no swelling of the polymer sample is observed but implantation profiles with maximum concentrations of 55% and 68% are obtained. For a diffusion coefficient of 1.67 × 10^−10^ cm^2^ s^−1^ the concentration is reduced to about 2% for both primary ion species and for even larger coefficients the Ne and Ar concentrations are almost reduced to 0. When comparing this to the experimental diffusion coefficients, which lie in between 1.67 × 10^−8^ and 1.67 × 10^−5^ cm^2^ s^−1^, it shows that almost no rare gas will accumulate in the polymers during He^+^, Ne^+^ and Ar^+^ bombardment. The out-diffusion of rare gas species during sample irradiation allows also to explain why no sample swelling has been observed in the experimental study. Compared to this, the diffusion coefficient for He in silicon at 300 K is of the order of 1.67 × 10^−16^ cm^2^ s^−1^ [36], hence several orders of magnitude below the values for polymers. Thus, sample swelling and blistering as well as bubble formation are only of concern for the analysis of inorganic samples by HIM. For polymer samples, the rare gas species can diffuse out of the sample and analysis results are not altered by this process. Figure 5 and Figure 6 show only the results for the ion irradiation of polyethylene, but the same observations have been made for the other polymers investigated in this work.

### Behaviour of polymers under helium, neon and argon bombardment

In this section, SD_TRIM_SP simulations are carried out with the diffusion coefficients shown in Table 1. The simulations are used to investigate several aspects which are of importance for the analysis of organic matter by HIM. They include the preferential sputtering which leads to the enrichment of some species compared to others, the evolution of these processes with fluence, impact energy and sample composition, and the damage induced by the ion bombardment inside the sample. The goal is to see to what extent analysis results get affected by the different processes, in particular for secondary ion imaging, and if some corrections need to be applied and, if yes, how they can be done.

**Figure 7:**
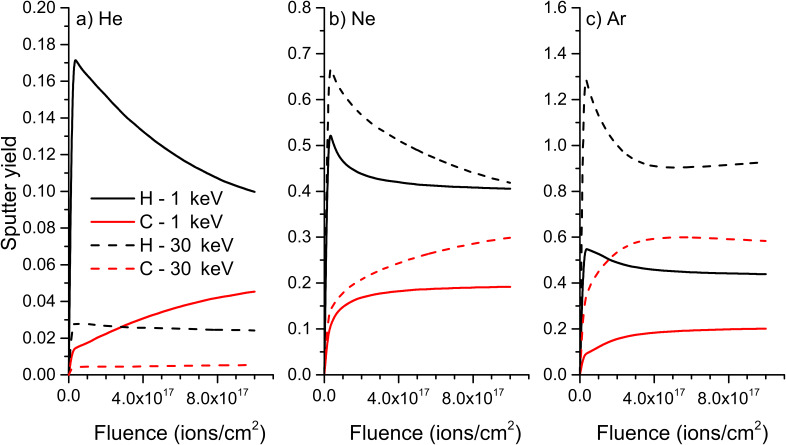
Evolution of sputter yields with fluence for a) helium, b) neon, and c) argon bombardment of PE at 1 keV and 30 keV.

At first, the evolution of sputter yields with fluence will be discussed. For 1 keV He^+^ bombardment of polyethylene, there is a huge preferential sputtering of hydrogen at the beginning of the process and even at a maximum fluence of 10^18^ ions/cm^2^, the equilibrium regime is still not reached, meaning that the sample surface continues to be depleted in hydrogen (Figure 7). For 30 keV He^+^, the preferential sputtering is far less pronounced and the system approaches equilibrium faster, i.e., it gets close to it for a fluence of about 8 × 10^17^ ions/cm^2^. The partial sputter yields are also higher at 1 keV than at 30 keV which is due to the increased implantation depth of helium to a point where the energy deposited close to the sample surface gets reduced and results in a lowering of the sputter yield. For the Ne and Ar bombardment, the trend is opposite. This is due to differences in interaction with sample atoms. For helium mainly electronic stopping is present while nuclear stopping is dominant for neon and argon. The overall stopping power is larger for the bigger species. With increasing impact energy the amount of energy deposited at the sample surface increases and leads to higher sputter yields. For these elements, the preferential sputtering is also more pronounced at higher impact energies and it takes also a higher fluence to get close to equilibrium for these conditions. The preferential sputtering is also less pronounced for argon than for neon, meaning that this process will be more of an issue for SIMS performed with He and Ne compared to more traditional SIMS primary ion species.

**Figure 8:**
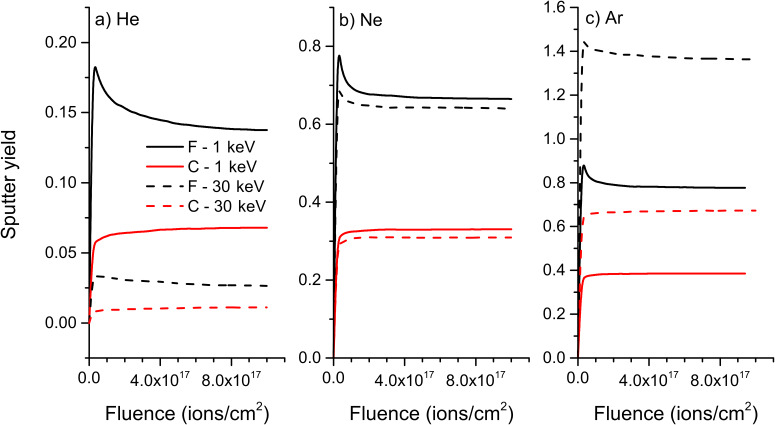
Evolution of sputter yields with fluence for a) helium, b) neon, and c) argon bombardment of PTFE at 1 keV and 30 keV.

The sample composition has also a huge influence on the preferential sputtering. When comparing results from PE (Figure 7) to PTFE (Figure 8), the reduced preferential sputtering for fluorine compared to hydrogen becomes obvious. Hence, it will be less of an issue for samples with heavier atoms or with atoms having a smaller difference in weight. The equilibrium regime with constant partial sputter yields is reached faster and the difference between maximum partial sputter yield for F and H at the beginning of the ion irradiation and the equilibrium partial sputter yield is smaller. The influence of the primary ion species remains however the same, the lowest preferential sputtering being observed for the heaviest primary ion and most pronounced being observed for helium.

**Figure 9:**
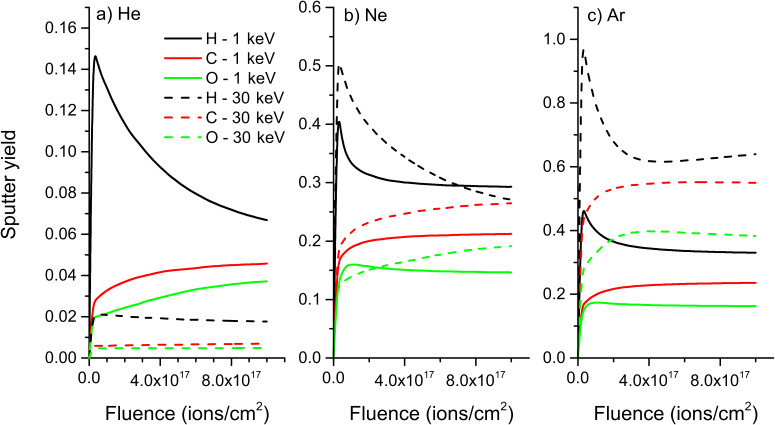
Evolution of sputter yields with fluence for a) helium, b) neon, and c) argon bombardment of PMMA at 1 keV and 30 keV.

For PMMA, which contains the three elements H, C and O, the influence of the mass of the elements is best seen (Figure 9), but the general trends are the same than for previous samples. The preferential sputtering of hydrogen is even more pronounced than for PE. At the same time, the partial sputter yield of carbon starts to increase at a lower fluence than the one of oxygen, leading to an enrichment of carbon compared to oxygen. This behaviour is also due to the difference in mass between the two species. The results for PS are not shown here, but the influence of the mass of the different elements on the sputtering process is the same.

**Figure 10:**
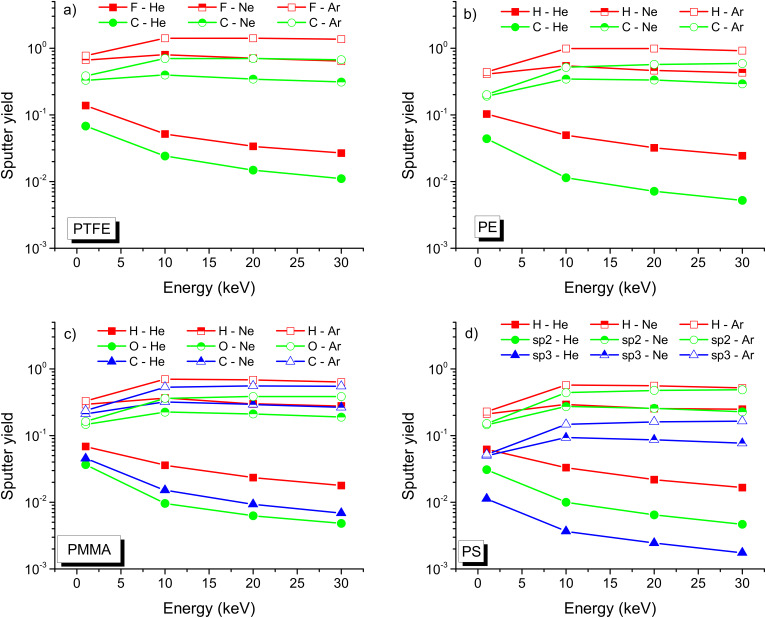
Partial sputter yields for the different chemical elements sputtered from a) PTFE, b) PE, c) PMMA, and d) PS for helium, neon and argon irradiation at a fluence of 10^18^ ions/cm^2^.

An overview of the different partial sputter yields for the energy range of 1–30 keV is given in Figure 10. For all conditions, the sputter yields under He^+^ irradiation are decreasing with impact energy. For Ne^+^, the sputter yields are constant over the investigated range of impact energies and for Ar^+^ they are only slightly increasing. The evolution of partial sputter yields reflects the amount of energy deposited close to the sample surface and which contributes to sputtering. The relation between the partial sputter yields of the different species is determined by the surface binding energies and the atomic number. F has the smallest surface binding energy (0.82eV), followed by H (1.00 eV), O (2.58 eV), C in sp^3^ configuration (5.00 eV) and C in sp^2^ configuration (7.37 eV) [25].

The difference in partial sputter yields leading to the preferential sputtering produces also an increase or lowering of the surface concentration of the different species. The surface concentrations stabilise normally at a fluence similar to where constant sputter yields are observed. However there is no direct correlation because changing sputter yields are also related to sample modification below the topmost 5 Å. For PE, the H and C surface concentrations are not stable at the maximum fluence of 10^18^ ions/cm^2^, which is in agreement with the evolution of the sputter yields. The same is true for 1 keV Ne and Ar bombardment. For 30 keV Ne and Ar irradiation, the concentrations remain constant after a fluence of about 5 × 10^17^ ions/cm^2^. For PTFE, the trend for the surface concentrations follows also the behaviour of the sputter yields, with stable concentrations after a fluence of 10^17^ ions/cm^2^ for Ne and Ar irradiation of 1 and 30 keV. For He, somewhat higher fluences of 4 × 10^17^ ions/cm^2^ at 1 keV and 10^18^ ions/cm^2^ at 30 keV are required. For PMMA with its three elements, the situation is slightly different and the concentration of one element might be stable while the two remaining concentrations are still changing. For 1 keV He, the C surface concentration is stable after a fluence of 4 × 10^17^ ions/cm^2^ while the H and O concentrations stabilise only after a fluence of 8 × 10^17^ ions/cm^2^. The same is observed at slight higher fluences for 30 keV He. For 1 keV Ne bombardment, the H concentration stabilises first at a fluence of 2 × 10^17^ ions/cm^2^ while C and O concentrations change up to a fluence of 4 × 10^17^ ions/cm^2^. For 30 keV Ne, the C concentration stabilises first at a fluence of 4 × 10^17^ ions/cm^2^ while the H and O concentrations change up to a fluence of 10^18^ ions/cm^2^. For 1 keV Ar, all surface concentrations stabilise at 4 × 10^17^ ions/cm^2^. For 30 keV the behaviour is the same than for 30 keV Ne. Hence, the impact energy and primary ion species have a significant influence on the surface composition and the flux of sputtered particles at a given fluence can differ significantly from the initial sample composition.

**Figure 11:**
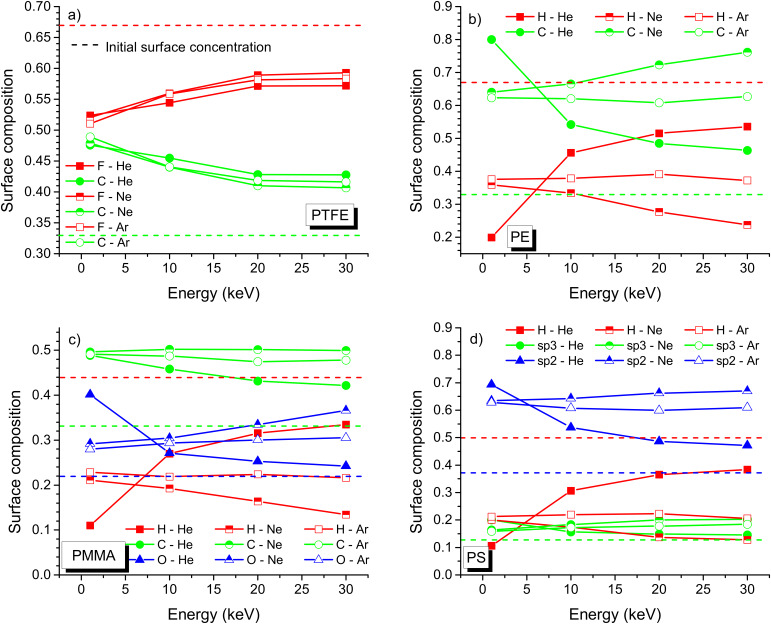
Surface composition as a function of impact energy for helium, neon and argon bombardment of a) F and C for PTFE, b) H and C for PE, c) H, C and O for PMMA, and d) H, C-sp^3^ and C-sp^2^ for PS. The results have been obtained by SD_TRIM_SP simulations for a fluence of 10^18^ ions/cm^2^.

On overview of the surface composition for different samples, primary ion species and impact energies at the end of the simulations, i.e., for a fluence of 10^18^ ions/cm^2^ is given in Figure 11. The surface composition is influenced a lot by the impact energy of the primary ions. At higher energies required for the high-resolution imaging, the preferential sputtering is more important than at the low energies used for depth profiling. For PTFE, the difference between initial sample composition and surface composition after a fluence of 10^18^ ions/cm^2^ is smallest at the highest impact energies (Figure 11a). A kind of plateau seems even to be reached at 30 keV. For these conditions, the sample modification is also smallest for He bombardment while the largest change is observed for Ne bombardment. Nevertheless, the differences in sample composition are rather small at high impact energies, with 43% carbon for He bombardment and 41% for Ne bombardment. At 1 keV, the differences are even much smaller with 49% of carbon for Ar bombardment and 47% of carbon for He bombardment. Compared to the initial sample composition is 33% of carbon and 67% of fluorine the enrichment in carbon and loss of fluorine are however important. The sample surface is also always enriched in carbon. Normally the sample is expected to get enriched in the heavier elements. The particular chemical properties of carbon and its ability to form chains may explain this behaviour. For PE too the sample surface gets enriched in carbon. However, here this behaviour is expected because of the small mass of hydrogen (Figure 11b). For PE, the initial sample composition is 33% of carbon and 67% of hydrogen. The absolute changes are higher than for PTFE, with a carbon concentration of 80% at 1 keV He bombardment and of 62% at 1 keV Ar bombardment. At high energies the carbon enrichment seems to be less for He bombardment, but increases for Ne and Ar bombardment. At 30 keV, a fluence of 10^18^ ions/cm^2^ produces a carbon surface concentration of 46% for He bombardment and of 76% for Ne bombardment. The concentration for Ar is in between. The extreme enrichment in carbon at low energy He bombardment and its decrease at higher energies is probably due to steady-state conditions which are not reached at a fluence of 10^18^ ions/cm^2^ and to surface concentrations corresponding to transition state. No physical explanation could be found to support a completely different behaviour for He^+^ than for Ne^+^ or Ar^+^. Hence, a similar trend should be observed for all primary ion species once steady-state conditions are reached. For He^+^ irradiation of PMMA and PS the same explanation is valid. Overall, the presence of hydrogen leads to the largest changes in surface composition and the difference to bulk concentrations increases with impact energy, except for samples with species having a similar mass. The general changes with an increased surface concentration of carbon agree with XPS data obtained on plasma-exposed PTFE [43].

Overall, for PS and PMMA the general trends agree with those observed for PE and PTFE (Figure 11c and d). A significant decrease in surface concentration with impact energy under He^+^ is observed for oxygen, i.e., the heaviest element. This agrees with the PE results However, in general the highest enrichment at the sample surface is observed for carbon, which agrees with the PTFE data and is most probably related to the chemical properties of carbon. For PS, the trends are very similar to PMMA, except that the changes in surface concentration for sp^3^-bonded carbon are small and sp^2^-bonded carbon acts as the heavier element. This makes also sense because it is more strongly bonded to other atoms. The degradation of polymer chains and the change of sp^3^ to sp^2^ carbon are not taken into account in SD_TRIM_SP simulations, so that only general trends are obtained. The actual concentration of both species could change. Identically, the hybridisation of some carbon atoms certainly changes for the other polymers, but cannot be simulated by SD_TRIM_SP. This would require the use of MD simulations which are too slow for simulations up to a fluence of 10^18^ ions/cm^2^.

**Figure 12:**
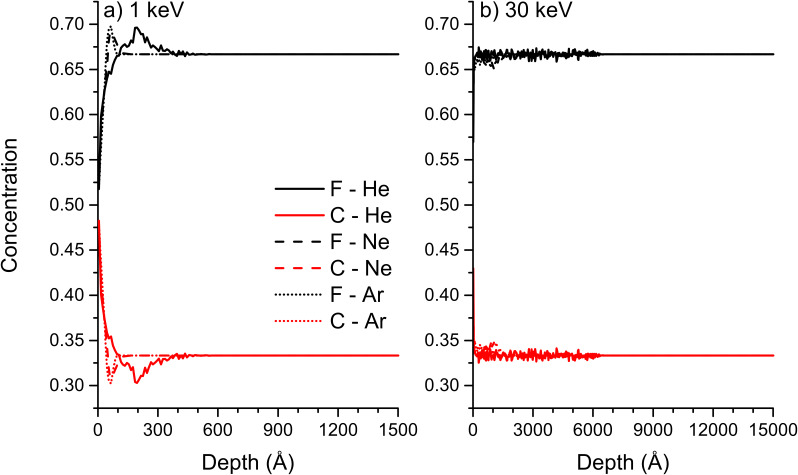
Concentration profiles of F and C at a fluence of 10^18^ ions/cm^2^ for He, Ne and Ar bombardment of PTFE: a) for 1 keV, and b) 30 keV impact energy. The results have been obtained by SD_TRIM_SP.

**Figure 13:**
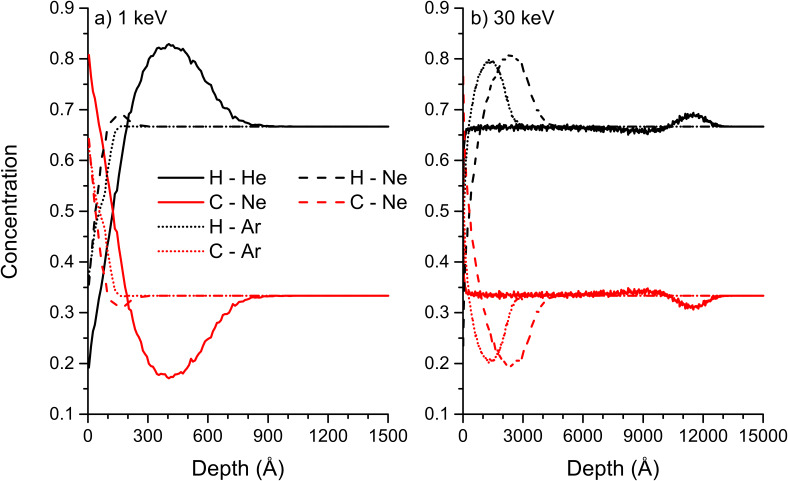
Concentration profiles of H and C at a fluence of 10^18^ ions/cm^2^ for He, Ne and Ar bombardment of PE: a) for 1 keV, and b) for 30 keV impact energy. The results have been obtained by SD_TRIM_SP.

Compared to data found in literature, the trends for surface concentrations of the different species agree qualitatively. For PMMA irradiated with 4 keV Ar^+^ ions, XPS gives a ratio of carbon to oxygen of 9 indicating a large enrichment of carbon compared to oxygen. This ratio is larger than the factor 2 predicted by SD_TRIM_SP, but in both situations the largest enrichment is obtained for carbon [44]. Hydrogen cannot be detected by XPS, so no comparison is possible for this element. In general, in SD_TRIM_SP sputtering depends on the surface binding energy which is calculated depending on sample composition, but the emission of volatile compounds and chemical reactions taking place in the sample under ion irradiation are not taken into account. The latter include reactions between oxygen and carbon as well as the change of hybridisation for carbon. These different aspects can lead to some discrepancy between experimental and simulated values. For PS, this becomes apparent for the increase of the C sp^2^ concentration compared to the C sp^3^ concentration due to the lower binding energy of the latter. Experimentally, the opposite is observed [45–46]. This can be explained by the inability of SD_TRIM_SP to change the hybridisation of a given element. However, hydrogen concentrations around 20% and global carbon concentrations are in good agreement with experimental findings [47]. The polymer samples are not only modified at the surface, but the rare gas irradiation induced also damage in the bulk of the material. The implantation at different energies for a fluence of 10^18^ ions/cm^2^ is shown for PTFE and PE (Figure 12 and Figure 13). For PTFE at 1 keV, the sample composition is changed most at the maximum implantation depth for He. For Ne and Ar bombardment the change in sample composition is comparable and far less than for He. It is also much closer to the sample surface due to the larger size of the primary ions. For Ne and Ar at 30 keV, no major change in sample composition can be observed, but some changes are observed down to a depth of 600 nm for He bombardment. The implantation probably goes along with chain scission and other damage, which cannot be verified by SD_TRIM_SP simulations. For PE, the difference in mass of the sample species produces a much larger change in concentration for He. At 1 keV, the hydrogen concentration increases above 80% at the main implantation depth of helium. This is opposite to the sample surface concentration which is largely enriched in carbon. A similar behaviour is observed for Ne and Ar bombardment, but it decreases with the mass of the primary ion. At 30 keV the implantation depth is largely increased, leading to a smaller change in composition for He bombardment. Only a small enrichment in hydrogen is observed at the maximum implantation depth. For Ne and Ar bombardment, the changes are more important and hydrogen concentrations at the maximum implantation depth are comparable to the hydrogen concentration at 1 keV He bombardment. Results for PS and PMMA are not shown but they are similar to PE and PTFE.

## Conclusion

Roughness and swelling is no major issue for polymers under He^+^, Ne^+^, or Ar^+^ irradiation in the energy range of 1 to 30 keV. The same should be true for higher energies in the keV range. Opposite to what is observed for inorganic materials, the rare gas atoms can diffuse out of the polymer samples, allowing only a small number of rare gas atoms to accumulate in the sample and lead to concentrations which can produce swelling or bubble formation. This is supported by MD simulations which show that diffusion coefficients are several orders of magnitude higher for organic materials than for inorganic ones. Hence, the typical Gaussian-shaped implantation profile is not observed for the rare gas primary ions. This does not mean that the He^+^, Ne^+^ and Ar^+^ bombardment do not produce any major modification of the polymer samples. There is an important preferential sputtering of hydrogen compared to heavier sample species, leading to an enrichment of carbon at the sample surface up to 80%. This effect is more pronounced for He than for Ne or Argon where maximum surface concentrations of carbon of 75% and 63% are observed. For Ne and Ar bombardment, equilibrium concentrations are already reached at a fluence of 10^18^ ions/cm^2^. For He irradiation, steady state conditions are not yet attained at this maximum fluence simulated in this work. The highest preferential sputtering is also observed for the high impact energies used in imaging applications, except for helium where the high implantation depth leads to low preferential sputtering in combination with extremely low sputter yields. The damage by rare gas irradiation is not limited to the near-surface area, but major modifications in sample compositions are observed close to the maximum implantation depth, i.e., in a region where the stopping power is highest. A more detailed understanding of polymer damage under He or Ne irradiation can only be obtained by MD simulations. Nevertheless, already now it is clear that the preferential sputtering and possibly the subsurface damage need to be taken into account when analysing secondary ion images obtained by ion microscopy. The situation is complicated by the fact that no systematic change for the dependence of the sample surface concentration on the primary ion species, impact energy and sample composition can be found.

## Methods

### Experimental setup

For this study, different polymer samples were spin-coated on silicon (111). The homopolymers of polystyrene (PS) with molecular mass *M*_w_ = 1 900 (*M*_w_/*M*_n_ = 1.10) and poly(methyl methacrylate) (PMMA) with molecular mass *M*_n_ = 22 200 (*M*_w_/*M*_n_ = 1.07) were obtained from Sigma-Aldrich. For PS toluene was used as a solvent and PMMA was diluted using chloroform as a solvent. Before spin-casting, the polymers were diluted in their respective solvents at between 10 wt % and 2 wt %, respectively. Thereafter, the polymer solution was deposited onto the cleaned wafer. The spin-casted parameters were 10 000 rpm/s spinning acceleration and 2 000 rpm spinning speed for a time period of 60 s. For this sample preparation a SPIN150-v3 spincoater was used.

The sputtering was performed on a Cameca IMS 4f [48]. The primary ions were generated in a cold cathode duoplasmatron ion source from Cameca [49], For the generation of the He^+^ primary ion current, a 20:80 percent Ne/He gas mixture was needed. The He^+^ and Ne^+^ beam were then selected using the magnetic sector on the primary column. As stated in [50], the primary ion beam current (20–100 nA) and diameter (25–100 µm) depended on the primary ion species. Irradiation of polymer samples was performed with 5.5 and 14.5 keV Ne^+^ and He^+^ primary ions, resulting in 25° and 36° incidence with respect to the surface normal. The primary ion beam was raster-scanned over a surface of 250 × 250 μm^2^.

On the PS and PMMA samples, the fluence was progressively increased from 10^15^ to 10^18^ ions/cm^2^. Using a current of 1 pA and a pixel size of 2 nm on the helium ion microscope (HIM), this would correspond to dwell times between 6 µs and 6 ms. The latter is much longer than dwell times used for secondary electron imaging on the HIM, but can be reached for multi-frame secondary ion images. Thereafter, AFM measurements in tapping mode (AFM probe type: ACST soft tapping mode tips without coating from Appnano) on an Agilent 5100 Surface Probe Microscope [51] were carried out inside the different post-bombardment craters in order to measure the topography as well as the roughness. In order to be able to compare the roughness measured at the bottom of the different craters, all AFM images consisted of 512 × 512 pixels corresponding to an area of 1 × 1 μm^2^. The RMS roughness was calculated using the Gwyddion software tool [52]. The average roughness measurement is based on the roughness from 6 different linescans covering different areas of the scanned image.

### Numerical methods

#### Diffusion of rare gas atoms

To study the mobility of noble gas atoms in a polymer matrix molecular dynamics (MD) simulations have been used to track the time evolution of the He, Ne and Ar positions in the system at atomic level. Four different polymers have been selected: polyethylene (PE), polytetrafluoroethylene (PTFE), atactic polystyrene (PS) and poly(methyl methacrylate) (PMMA). Different polymer samples have been created, each sample containing 10 molecules. To get samples of similar size, each molecule contains 100 carbon atoms, except for PS molecules which contain 104 carbon atoms. The total number of atoms (3020) is equal for the PE, PMMA and PTFE samples, while the PS systems contain 2100 atoms. The different samples have been generated using the Polymer Modeller software [53] with the initial equilibration being carried out at constant number, volume and temperature (NVT) in LAMMPS [54] with the Dreiding force field [55]. The size of the systems is about 3 × 3 × 3 nm^3^ and the initial density has been set to reflect the real low- (LD) and high-density (HD) states of each polymer (Table 2). More detailed information can be found in Table 1. The temperature was set to 300 K.

**Table 2:** Sample densities for this work and reference values.

system	density [g/cm^3^]					
	ultra-low density		low density (LD)		high density (HD)	
	this work	reference	this work	reference	this work	reference

PE	0.86	0.86 [56–58]	0.92	0.92 [59–60]	0.96	0.96 [59–60]
PS		—	1.01	0.99 [61]	1.04	1.05 [61]
PMMA		—	1.17	—	1.20	1.19 [42]
PTFE		—	1.55	1.55 [62]	2.17	2.17 [62]

Next, 5 noble gas atoms (He, Ne or Ar) have been added to the different polymer samples to study their diffusion behaviour. The initial positions of the rare gas atoms have been set randomly with the restriction that the distance to the closest polymer atom is larger than 2.5 Å and the distance to the closest rare gas atom is larger than 7.5 Å. This ensured that the concentration of the noble gas atoms inside the polymer samples was low enough to reduce the possibility of collective motion of several diffusing atoms, which might artificially increase the diffusion coefficient. To increase statistics, two samples of each polymer–rare gas combination have been created. Afterwards, the final equilibration has been carried out using Gromacs [63] with the Opls-AA force field [64]. The samples have been kept at 300 K for 5 ns using NVT conditions with the temperature controlled by the Berendsen thermostat [65] with a coupling constant of 0.1 ps. Afterwards the samples were heated up to 600 K with a speed of 1 K/ps and kept at this temperature for 1 ns before cooling down to 300 K with the same cooling rate than for heating and keeping them at 300 K for 5 ns from which the last 4.8 ns were used to study the diffusion of the rare gas atoms. The NVT algorithm has been used to preserve the density of the samples.

#### Sputtering processes

Simulations on sputtering were carried out using the SD_TRIM_SP code [25] which is based on the simulation codes TRIM [66–67] and TRIDYN [68–69]. In addition to previous codes, SD_TRIM_SP includes the option to take the outgassing of atoms in a sample into account [70]. This is required for the simulation of helium and neon bombardment in polymers. The KrC potential has been used for interatomic interactions, the Oen–Robinson model for electronic stopping and the Gauss–Mehler method with 16 pivots for integration. The surface binding energy is calculated using *sbe*(*i*,*j*) = 0.5(*Es_i_* + *Es_j_*), where *sbe* is the surface binding energy for the target of consideration and *Es_i_* is the atomic surface binding energy [25].

In a first step, the diffusion coefficients of the rare gas species have been changed in order to study the influence of the outgassing on the simulation results. In a second step, simulations on the sputtering of polymers have been carried out using the diffusion coefficients corresponding to experimental conditions.

To study the influence of polymer composition on the sputtering and outgassing processes, four different polymers have been investigated, polyethylene, polystyrene, polytetrafluoroethylene and poly(methyl methacrylate). Comparison to more conventional SIMS conditions is obtained by using argon bombardment in addition to helium and neon bombardment. The impact energies of 1 keV to 30 keV have been chosen to cover the conditions for depth profiling and imaging.
